# Correction: Malignancies in patients with inborn errors of immunity: insights from 20-years of clinical experience in Qatar

**DOI:** 10.3389/fimmu.2026.2010725

**Published:** 2026-10-09

**Authors:** Sally Mahgoub Khalil, Mehdi Adeli, Sara Mohamed, Sherin Thalappil, Salma A. Taha, Amna Gamil, Honar Charif, Tawfeg Ben-Omran, Mohammed S. Ehlayel, Maryam Ali Al-Nesf

**Affiliations:** 1 Corporate Allergy and Immunology Division, Department of Medicine, Hamad Medical Corporation, Doha, Qatar; 2 Department of Pediatrics, Pediatric Allergy, Hamad General Hospital, Hamad Medical Corporation, Doha, Qatar; 3 Department of Hematology, National Center for Cancer Care and Research, Hamad Medical Corporation, Doha, Qatar; 4 Weill Cornell Medical College, Doha, Qatar; 5 Qatar University, College of Medicine, Doha, Qatar; 6 Department of Pediatric Medicine, Sidra Medicine, Doha, Qatar

**Keywords:** ataxia telangiectasia, cancer, inborn errors of immunity, malignancy, primary immunodeficiency

The funder Medical Research Centre, Hamad Medical Corporation, MRC-01-25-141|, for Sally Mahgoub Khalil was erroneously omitted. The original version of this article has been updated.

